# The Safety Evaluation of Branched-Chain Fatty Acid Derived from Lanolin and Its Effects on the Growth Performance, Antioxidant, Immune Function, and Intestinal Microbiota of C57BL/6J Mice

**DOI:** 10.3390/nu18020351

**Published:** 2026-01-21

**Authors:** Jingyi Lv, Yang Cao, Yibo Zhu, Haitao Du, Chunwei Wang, Weiguo Ding, Huihuan Liu, Hangshu Xin, Guangning Zhang

**Affiliations:** 1 College of Animal Science and Technology, Northeast Agricultural University, Harbin 150030, China; b210501015@neau.edu.cn (J.L.);; 2 Heilongjiang Provincial Dairy Industry Association, Harbin 150020, China; 3 Heilongjiang Provincial Animal Husbandry General Station, Harbin 150069, China; 4 United Bio-Tech Co., Ltd., Guangzhou 511356, China

**Keywords:** branched-chain fatty acids derived from lanolin, acute toxicity, growth performance, blood metabolism, intestinal microbiota

## Abstract

**Background/Objectives**: Branched-chain fatty acids (BCFAs) exhibit a range of biological activities; however, their limited natural abundance and high cost have constrained in vivo research. Lanolin represents a promising source for enriching BCFAs. Nevertheless, the in vivo application, safety, and dose-effect relationship of BCFAs derived from lanolin (BCFAs-DFL) remain unassessed. **Methods**: In this study, the acute toxicity in C57BL/6J mice was first evaluated for 7 days by a single oral administration of 5000 mg/kg BW of BCFAs-DFL. Subsequently, 40 mice were divided into four groups (control group, low dose of 100 mg/kg BW, medium dose of 300 mg/kg BW, and high dose of 600 mg/kg BW) and were continuously administered by gavage for 28 days to study the effects of BCFAs-DFL on the growth, blood biochemistry, intestinal morphology, and intestinal flora of the mice. **Results**: In the acute toxicity test, BCFAs-DFL exhibited no lethality or abnormalities in mice, indicating its non-toxic nature. Throughout the 28-day trial, mice in the medium- and high-dose groups experienced a notable decrease in average daily feed intake (*p* < 0.05), yet their weight gain remained unaffected (*p* > 0.05). Hemoglobin and hematocrit levels declined in the high-dose group (*p* < 0.05). Conversely, serum aspartate aminotransferase and total bilirubin levels escalated in the medium- and high-dose groups, while triglycerides and urea nitrogen levels decreased (*p* < 0.05). The serum’s total antioxidant capacity and immunoglobulin levels (IgA, IgG) rose in proportion to the dosage (*p* < 0.05). BCFAs-DFL notably enhanced the villus height of the jejunum and ileum in mice (*p* < 0.05). Gut microbiota analysis indicated no significant impact on overall α and β diversity. **Conclusions**: The 28-day intervention revealed that BCFAs-DFL can modulate feeding behavior, TG, T-AOC, and immunoglobulin levels in mice. Additionally, it promotes the development of intestinal villi. Based on various indicators, a dosage of 100 mg/kg BW effectively induces beneficial metabolic regulation, such as the reduction of triglycerides, without causing a burden on liver metabolism. This dosage may represent a more suitable application for potential use.

## 1. Introduction

Branched-chain fatty acids (BCFAs) are a class of lipids characterized by the presence of one or more methyl branches attached to the alkyl chain, typically at the second (iso-) or third (anteiso-) carbon position [1]. These compounds exhibit various bioactivities, including anti-inflammatory, lipid-lowering, and anticancer properties [2]. Notably, approximately 30% of the lipids in neonatal vernix caseosa contain BCFAs, which can be continuously absorbed by the fetus through amniotic fluid during gestation. This may partly explain the higher susceptibility of preterm infants to necrotizing enterocolitis (NEC) compared to term infants [3,4,5]. Supporting this, feeding preterm rat models with a lipid mixture mimicking the BCFA composition of fetal lipids—specifically containing iso-14:0 (25%), anteiso-C15:0 (20%), iso-16:0 (25%), anteiso-C17:0 (8%), iso-18:0 (10%), and iso-20:0 (12%)—reduces NEC incidence by 56% and significantly upregulates IL-10 gene expression in ileal mucosa, along with increasing the relative abundance of *Bacillus subtilis* [1]. Intriguingly, lipids from *Bacillus subtilis* contain approximately 90% BCFAs and are regarded as a symbiotic source associated with BCFAs activity [1]. However, it remains unclear whether these protective effects stem from elevated dietary BCFA levels or enhanced immune modulation. BCFAs also play a role in metabolic health. Serum levels of iso-BCFAs are lower in obese individuals compared to healthy controls [6]. It has also been reported that a high daily intake of BCFA-rich foods is associated with a reduced risk of cardiovascular and metabolic diseases [7]. In mice fed a high-fat diet, BCFAs extracted from yak butter olein reduced serum cholesterol via the downregulation of the HMGCR expression [8]. In vitro studies showed that treatment of human hepatocytes (L02) with iso-C15:0 and iso-C18:0 decreased intracellular triglyceride accumulation [9]. Moreover, BCFAs have attracted considerable attention in cancer research. Iso- and anteiso-forms such as C15:0 and C17:0 are reported to inhibit cancer cell proliferation and induce apoptosis, with iso-C15:0 and anteiso-C15:0 exhibiting particularly pronounced effects, whereas iso-C14:0, iso-C16:0, and iso-C18:0 tend to exert cytotoxic activity [10,11].

Most existing studies on BCFAs are clinical or metabolic in nature, with mechanistic insights largely derived from cell-based models. In vivo studies, particularly in mice, remain limited [8,12,13], partly due to the low natural abundance and high cost of BCFAs. Although BCFAs occur widely in nature, their concentrations are generally low. They were initially identified as structural components of microbial membranes such as *Bacillus subtilis* [1,14] and are found in fermented foods (e.g., natto and miso) and ruminant products, typically accounting for less than 2% of total fatty acids [2,15]. Aside from neonatal vernix caseosa, lanolin-a byproduct of wool processing-has recently been identified as a rich source of BCFAs, with contents reaching up to 50% of total fatty acids, the highest known natural concentration [16,17]. Although microbial biosynthesis and chemical synthesis routes have been explored, these methods often yield complex byproduct profiles or involve hazardous reagents, limiting their application in pharmaceutical and industrial fields [18,19]. A promising alternative is the enrichment of BCFAs from lanolin using urea inclusion technology, as demonstrated by Wang et al. [16], which may facilitate future in vivo and animal studies.

To date, no reports have evaluated the in vivo application of BCFAs derived from lanolin (BCFAs-DFL). Consequently, their safety profiles and appropriate dosages remain unexplored. In this study, we utilized C57BL/6J mice as model organisms to initially assess their safety. We then investigated the effects of varying doses on growth, metabolism, intestinal development, and microbiota composition in the mice, ultimately identifying an appropriate application dose. This research offers insights into the functions and dosages of BCFAs-DFL for potential future applications in biological systems.

## 2. Materials and Methods

### 2.1. Preparation of BCFAs-DFL and Emulsion

The BCFAs were extracted from lanolin using the method described by Wang et al. [16]. Briefly, lanolin was first saponified at 70 °C for 4~6 h using an ethanolic sodium hydroxide solution and then converted into calcium fatty acid salts via reaction with calcium chloride. The calcium salts were repeatedly washed with anhydrous ethanol under heating to remove unsaponified lanolin alcohols. After ethanol washing, the calcium salts were acidified with hydrochloric acid, and the liberated fatty acids were extracted with n-hexane and concentrated by vacuum distillation to obtain crude lanolin fatty acids. Subsequently, the obtained lanolin fatty acids were mixed with urea and 95% ethanol in a ratio of 1:2:6 (*m*/*m*/*v*) and allowed to react at 60 °C for 2 h. The mixture was cooled to room temperature and kept at 4 °C for 12 h to promote the formation of urea inclusion crystals. The crystals were thoroughly separated and washed multiple times with ethanol. The liquid phase was concentrated by vacuum distillation to yield a urea–fatty acid mixture. This product was then completely dissolved in a 5% sodium chloride solution to dissolve the urea, and the fatty acids were finally extracted with petroleum ether to obtain BCFAs-DFL. Methyl esterification of fatty acids was performed according to the methods described by Xin et al. [20]. The composition of BCFAs-DFL was analyzed using a Shimadzu Nexis GC-MS system (GC2030/QP2020NX, Kyoto, Japan). All derivative samples were injected into the GC-MS system and separated using a DB-5 capillary column (30 m × 0.25 mm × 0.25 μm, Agilent, Santa Clara, CA, USA). Chromatograph acquisition and mass spectrometry identification were performed by chromatography–mass spectrometry software (LabSolutions, Shimadzu, Kyoto, Japan). The chemical structure of each fatty acid was confirmed by searching the NIST spectrum library through the chemical workstation data processing system for spectrum analysis [21,22]. Finally, the area normalization method was used for the quantification of fatty acids. The composition of the resulting BCFAs-DFL is presented in Table 1.

Due to the poor water solubility of BCFAs-DFL at room temperature, direct administration to mice was not feasible. In accordance with common practices for delivering hydrophobic components (e.g., fish oil) in animal studies [23,24], an emulsion was prepared. In this study, soy lecithin was used as the emulsifier. In the acute toxicity test, BCFAs-DFL and the emulsifier were mixed at a mass ratio of 10:1. For the treatment groups (100, 300, and 600 mg BCFAs-DFL/kg BW), the corresponding mass ratios of BCFAs-DFL to emulsifier were 5:3, 5:1, and 10:1, respectively. In both experiments, the concentration of emulsifier in the control group was maintained at the same level as in the treatment groups. The mixture was then homogenized using a homogenizer (model FA30D, produced by Furuike Co., Ltd., Shanghai, China) at 15,000 rpm to form a uniform emulsion.

### 2.2. Experimental Animals, Design, and Sample Collection

All animal trials were conducted under a protocol approved by the Institutional Animal Care and Use Committee of Northeast Agricultural University (Approval No.: NEAUEC20240290). Special pathogen-free (SPF) C57BL/6J mice (male and female, aged 4–5 weeks and weighing 15–20 g) were obtained from Liaoning Changsheng Biotechnology Co., Ltd. (Benxi, China). All the animals were housed individually in sterile polypropylene cages under standard conditions: room temperature 24 ± 2 °C, humidity 55 ± 5%, and a 12 h light/dark cycle (lights on from 08:00 to 20:00). Male and female mice were kept separately with ad libitum access to water and a standard diet. A 7-day acclimation period was provided prior to experimental treatments.

The safety test was performed according to the experimental protocol Guideline 423 of the Organization for Economic Cooperation and Development (OECD) [25]. A total of 20 mice (10 females and 10 males) were randomly divided into the Control and BCFA groups, each group comprising 10 animals, of which 5 were male and 5 were female. The BCFA group received oral administration of 5000 mg/kg body weight (BW) of BCFAs-DFL, while the Control group received an equal volume of soy lecithin solution. All animals were observed daily for mortality and changes in general behavior (e.g., fighting, drowsiness, and abnormal excitement) for 7 days. Daily feed consumption was recorded. On day 8, all mice were weighed and then euthanized by cervical dislocation following deep anesthesia with chloral hydrate. Subsequently, the heart, liver, spleen, lungs, kidneys, and thymus were collected, weighed, and the coefficients of each organ were calculated using the following formula:

Organ coefficients (%) = (Organ weight)/(Body weight) × 100

Forty mice (20 males and 20 females) were used to investigate the effects of BCFAs-DFL on mouse growth, metabolism, immunity, and gut microbiota. The mice were randomly assigned to four groups (5 males and 5 females from each group): control (CON; vehicle), low-dose (low; BCFAs-DFL, 100 mg/kg BW), medium-dose (medium; BCFAs-DFL, 300 mg/kg BW), and high-dose (high; BCFAs-DFL, 600 mg/kg BW). Treatments were administered once daily for 28 consecutive days. The BW and abnormal behavior were observed daily during treatment. The weight was recorded weekly, and the consumption of feed was recorded daily. On d29, all mice were weighed, and blood samples were collected from the retroorbital plexus. Blood samples intended for hematological analysis were collected in EDTA-K_2_ tubes and stored at 4 °C until processing. For serum preparation, clotted blood samples were centrifuged at 3500 rpm for 15 min at 4 °C. The resulting serum was aliquoted and stored at −20 °C for subsequent assays. Mice were then immediately euthanized. The spleen and thymus were collected and weighed to calculate organ coefficients. Subsequently, approximately 1 cm of the jejunum and ileum were taken and placed in Carnoy’s fluid for histological examination. Additionally, cecal contents were collected, stored in 2 mL cryovials, flash-frozen in liquid nitrogen, and maintained at −80 °C until 16S rRNA sequencing analysis.

### 2.3. Hematological and Serum Parameters Analysis

Whole blood samples were analyzed for red blood cells (RBC), mean corpuscular volume (MCV), hematocrit (HCT), platelet crit (PCT), white blood cells (WBC), lymphocytes (LYM), granulocytes (GRA), and hemoglobin (HGB) using an automated hematology analyzer (Beijing Baolingman Sunshine Technology Co., Ltd.; Beijing, China).

Serum biochemical parameters such as total protein (TP), albumin (ALB), globulin (GLOB), aspartate transaminase (AST), alanine aminotransferase (ALT), total bilirubin (TBIL), triglyceride (TG), total cholesterol (TC), high-density lipoprotein cholesterol (HDLC), low-density lipoprotein cholesterol (LDLC), creatinine (CRE), blood urea nitrogen (BUN), and glucose (GLU) were analyzed using a biochemical analyzer (BS2000M2, Shenzhen, China). Catalase (CAT), glutathione peroxidase (GSH-PX), total antioxidant capability (T-AOC), and malondialdehyde (MDA) levels in serum were determined by following the manufacturer’s instructions for commercial colorimetric analysis kits (Nanjing Jiancheng Institute of Bioengineering; Nanjing, China). Serum immunoglobulin (Ig) A, IgG, and IgM concentrations were determined using commercial mouse ELISA kits (Jiangsu Jingmei Biotechnology Co., Ltd.; Yancheng, China).

### 2.4. Histopathological Evaluation

Tissue samples embedded in Carnoy’s fluid were washed with running tap water to remove residual fixative, followed by dehydration in a graded alcohol series (75%, 85%, 95%, and 100%). After clearing in xylene, tissues were infiltrated with molten paraffin wax. Embedding and sectioning were performed using a Leica embedding and microtome system (Leica, Wetzlar, Germany). Serial sections of 4 µm thickness were prepared and stained with hematoxylin and eosin (H&E). The intestinal morphology images were obtained through white light photography using a microscope (SWE-CX63; Servicebio, Wuhan, China). Whole-slide imaging was acquired using an EFL panoramic fully automated slide scanner (EFL, Suzhou, China) at appropriate magnifications, and the parameters of the intestinal villi and crypts in mice were measured using the ZYFViewer 2.0.1.10 medical image analysis software according to the protocol described by Kandelouei et al. (2024) and van Keulen et al. (2020) [26,27].

### 2.5. Cecal Contents Collection and 16S rRNA Gene Sequencing

Cecal contents were collected post-mortem from each mouse, immediately transferred into 2 mL sterile tubes, flash-frozen in liquid nitrogen, and stored at −80 °C until further analysis. From each treatment group, six samples (three females and three males) were randomly selected for microbial profiling. Genomic DNA was extracted from the cecal contents using the NucleoSpin 96 Soi DNA Kit (Macherey-Nagel, Düsseldorf, Germany) following the manufacturer’s instructions. The bacterial V3–V4 hypervariable regions were amplified using the primer pairs 314F (5′-CCTAYGGGRBGCASCAG-3′) and 806R (5′-GGACTACNNGGGTATATAAT-3′). To enable multiplexing, 6 bp barcode sequences were individually appended to the 5′ ends of both the forward and reverse primers. PCR amplification was performed in two steps: the first PCR involved thermal cycling with an initial denaturation at 95 °C for 5 min, followed by 30 cycles of 95 °C for 1 min, 60 °C for 1 min, and 72 °C for 1 min, with a final extension at 72 °C for 7 min; the second PCR, used to attach sample-specific barcodes, included an initial denaturation at 95 °C for 5 min, then 12 cycles of 95 °C for 1 min, 60 °C for 1 min, and 72 °C for 1 min, with a final extension at 72 °C for 7 min. PCR products were purified using AMPure XP (Beckman, CA, USA) beads and quantified via Qubit 3.0 fluorometry. High-throughput sequencing was performed on the Illumina NovaSeq 6000 platform (Novogene, Beijing, China) with paired-end PE250 chemistry. Raw sequencing data were processed using QIIME2 (2022.08) software [28]. Sequences were quality-filtered, denoised, assembled, and checked for chimeras using DADA2 (1.26) to generate amplicon sequence variants (ASVs) [29]. Taxonomic assignment of ASVs was performed by aligning sequences against the SILVA database (Release 138, https://www.arb-silva.de/documentation/release-138/, accessed on 10 January 2024) at a 99% sequence similarity threshold to obtain microbial taxonomic assignments at the genus and species levels. Additionally, α-diversity and β-diversity analyses were conducted within QIIME2 (2022.08) to evaluate microbial diversity and community structure.

### 2.6. Statistical Analysis

All data were assessed for normality prior to statistical analysis using the UNIVARIATE procedure of SAS 9.4 (SAS Institute Inc., Cary, NC, USA). Safety trial outcomes were analyzed using a *t*-test with the PROC TTEST procedure in SAS software, with a significance threshold set at *p* ≤ 0.050. In the experiment to explore the effects of different doses of BCFAs-DFL on the growth and metabolism of mice, data on growth performance, organ coefficients, hematological parameters, serum metabolites, and intestinal morphology were analyzed by analysis of variance (ANOVA) using the PROC MIXED procedure in SAS software. The model included treatment and sex groups, as well as their interaction (treatment × sex), as fixed effects, with mice as a random effect. Data on gut microbiota did not include a sex effect. Orthogonal polynomials for unequally spaced treatment levels were generated using the PROC IML procedure in SAS software. Linear, quadratic, and cubic trends were tested using the CONTRAST statement. Post hoc comparisons among group means were performed using Tukey’s test, with statistical significance defined as *p* ≤ 0.050. Results are presented as mean ± standard deviation (SD). The likelihood ratio test method of DESeq2 was used to test the significance of the differences among different treatments, and multiple comparisons were conducted using false discovery rate (FDR). A *p*-value ≤ 0.05 indicates a significant difference. The Kruskal–Wallis test was used to analyze indicators of α diversity. Alpha diversity indices were visualized using GraphPad Prism 9 (GraphPad Software Inc., San Diego, CA, USA). Using the reshape2, dplyr, tidyr, and ggplot2 packages in R software (version 4.4.3; https://www.r-project.org, accessed on 9 January 2026), a Spearman rank correlation analysis was conducted to examine the relative abundance of metabolites in mouse serum and bacterial genera in the cecal contents. *p*-values were adjusted for multiple comparisons using the FDR. Statistical significance was defined as an adjusted *p*-value (FDR) < 0.05, while an absolute Spearman’s correlation coefficient (|ρ|) > 0.7 was considered indicative of a strong correlation, where ρ is defined as the Spearman rank-order correlation coefficient [30].

## 3. Results

### 3.1. Acute Toxicity Induced by BCFAs-DFL in Mice

**Figure 1:**
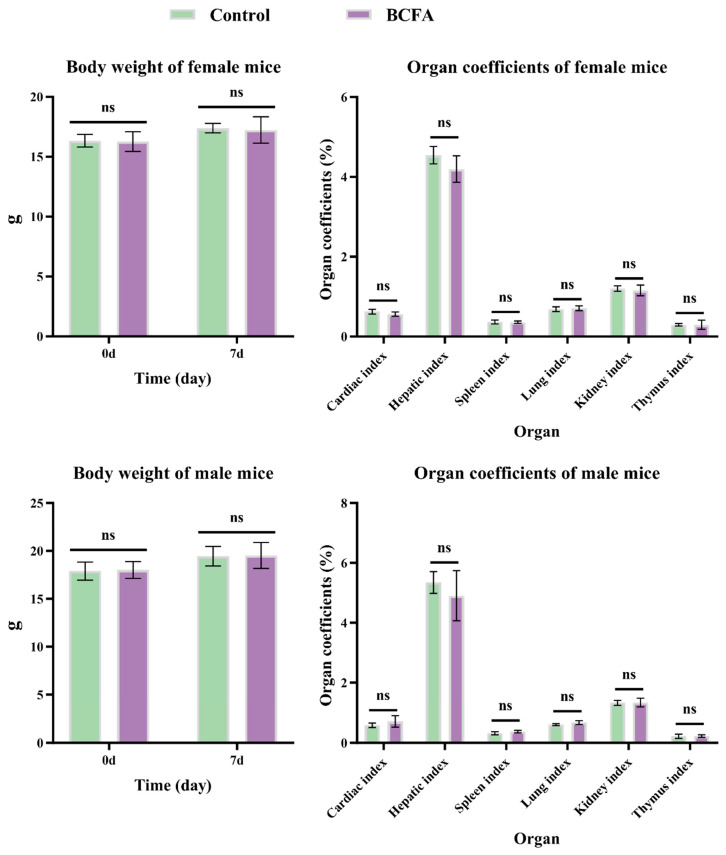
Body weight and organ coefficient results of mice treated orally with BCFAs-DFL in the acute toxicity test. Data are means ± SD, ns = not significant, *p* > 0.050.

In the acute toxicity study, a single oral gavage dose of 5000 mg/kg BW of BCFAs-DFL was administered to mice, followed by a 7-day observation period. No mortality was observed throughout the study (Table 2). Additionally, there were no significant differences in feed intake or activity levels between the BCFA and control groups. As shown in Figure 1, BW measured prior to administration and at 7 days post-administration showed no significant differences between the BCFA and control groups in either female or male mice (*p* > 0.050). Organ coefficient analyses also revealed no significant differences between groups (*p* > 0.050). Collectively, these results suggest that BCFAs-DFL at a dose of 5000 mg/kg body weight does not induce acute toxicity in C57BL/6J mice. Based on established classification criteria for the acute toxicity of external chemical agents, BCFAs-DFL can be regarded as practically non-toxic. Therefore, it can be concluded that administration of BCFAs-DFL at this dose does not produce adverse effects in vivo.

### 3.2. Growth Performance and Immune Organ Coefficients

Growth performance and immune organ coefficients in mice were not influenced by the interactions of treatment and sex. Both initial and final mouse weights, along with ADG, demonstrated no significant differences across treatments (*p* > 0.050; Table 3). In contrast, the 28-day mouse weight and ADG revealed cubic (*p* = 0.048) and linear (*p* = 0.023) changes, respectively. Average daily feed intake (ADFI) was significantly reduced in mice receiving medium- and high-dose BCFAs-DFL (*p* < 0.001), with this reduction exhibiting both linear (*p* < 0.001) and cubic (*p* = 0.001) effects. While the coefficients for the spleen and thymus were unaffected by BCFAs-DFL, they did exhibit cubic (*p* = 0.049) and linear (*p* = 0.036) changes, respectively.

### 3.3. Hematological Parameters

BCFAs-DFL had no impact on WBC, LYM, RBC, and GRA levels in mouse blood. However, RBC content decreased linearly with increasing BCFAs-DFL dose (*p* < 0.050; Table 4). HGB, HCT, MCV, and PCT levels in blood were significantly influenced by the treatment (*p* < 0.050). HGB and HCT levels were notably lower in the high group (*p* = 0.002) and displayed linear (*p* < 0.010) and quadratic (*p* < 0.050) changes with increasing BCFAs-DFL dosage. Supplemented mice did not differ significantly from the control group in MCV and PCT levels (*p* > 0.050) but showed significant linear effects (*p* < 0.050). Additionally, PCT levels exhibited a cubic effect (*p* < 0.050). Moreover, all hematology indexes remained unaffected by interactions between treatment and sex (*p* > 0.050).

### 3.4. Serum Metabolites

**Figure 2:**
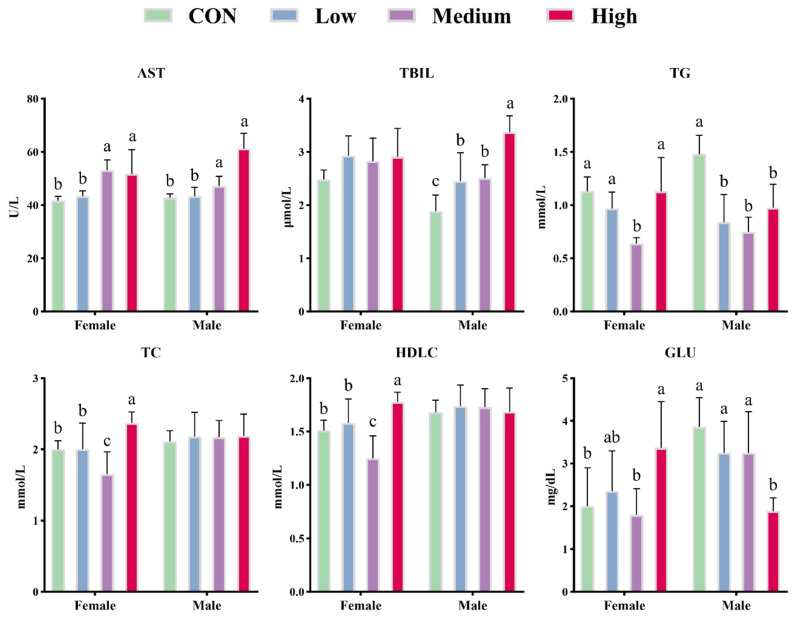
The effects of different doses of BCFAs-DFL on blood metabolites in female and male mice. Different letters (a–c) notation indicates a significant difference between treatments within the same gender (*p* < 0.05). The error bars represent the SD.

Supplementation with BCFAs-DFL did not significantly affect serum levels of TP, ALT, γ-GT, LDLC, GLOB, or GLU in mice (Table 5). However, serum LDLC levels exhibited a quadratic effect (*p* = 0.024) with increasing BCFAs-DFL dosage. Additionally, ALB and GLOB levels in the low- and high-dose groups were significantly lower and higher than those in the CON group, respectively (*p* < 0.050). The decrease in ALB levels demonstrated linear and cubic trends, whereas GLOB levels showed quadratic and cubic effects (*p* < 0.050). Serum AST levels were significantly elevated in the medium- and high-dose groups and followed a linear pattern (*p* < 0.001). Furthermore, BCFAs-DFL significantly increased serum TBIL levels, with linear (*p* < 0.001) and cubic (*p* = 0.035) effects observed. Serum TG levels significantly decreased in a linear (*p* = 0.048) and quadratic (*p* < 0.001) manner, with the medium-dose group exhibiting the lowest levels among the BCFAs-DFL-treated groups, all of which were significantly lower than the control (*p* < 0.001). Conversely, BCFAs-DFL did not significantly influence serum TC or HDLC levels (*p* > 0.050), although a quadratic trend was noted (*p* < 0.050). Serum UN levels displayed decreasing linear and quadratic trends (*p* = 0.001 and *p* = 0.049, respectively), and inter-treatment comparisons revealed significantly lower UN levels in the medium and high groups compared to the CON group (*p* < 0.050). Moreover, serum levels of AST, TBIL, TG, TC, HDLC, and GLU were influenced by interactions between treatment and sex (*p* < 0.050; Figure 2). Specifically, AST levels were significantly elevated in both female and male mice subjected to the medium and high doses (*p* < 0.050). BCFAs-DFL increased serum TBIL levels only in male mice (*p* < 0.050). In males, TG levels decreased significantly across all treated groups, whereas in females, only the medium dose resulted in a significant reduction (*p* < 0.050). Serum TC and HDLC levels were affected by BCFAs-DFL exclusively in females, with the medium and high doses inducing significant decreases and increases, respectively (*p* < 0.050). Notably, high-dose BCFAs-DFL significantly elevated serum glucose levels in female mice but had the opposite effect in males (*p* < 0.050).

Levels of serum GSH-PX and MDA remained unaffected by BCFAs-DFL treatment (Table 5). However, CAT activity decreased linearly, while T-AOC increased linearly with higher BCFAs-DFL doses (*p* < 0.050). Serum CAT levels did not differ significantly between the control and BCFAs-DFL groups, although the low-dose group showed a numerically higher value (*p* > 0.050). Importantly, BCFAs-DFL supplementation resulted in a significant elevation of serum T-AOC levels (*p* < 0.050). BCFAs-DFL significantly increased serum IgA and IgG levels (*p* < 0.001). With increasing BCFAs-DFL supplementation doses, IgA content exhibited quadratic (*p* < 0.001) and cubic (*p* < 0.001) effects, while IgG levels showed linear (*p* = 0.034) and quadratic (*p* < 0.001) effects. Serum IgM content remained unaffected by treatment (*p* > 0.050).

### 3.5. Intestinal Morphology

**Figure 3:**
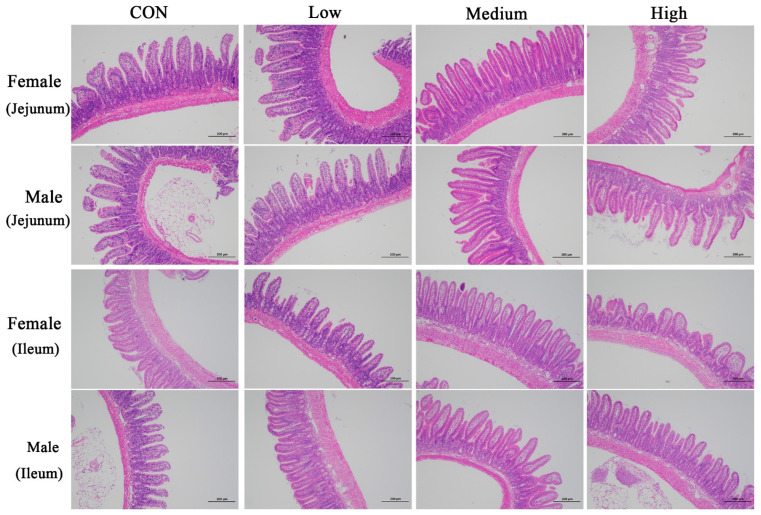
Light microscopy (100×) of the intestinal morphology in the jejunum and ileum of mice.

BCFAs-DFL administration significantly increased villus height in both the jejunum and ileum of mice (*p* < 0.001; Table 6 and Figure 3), without influencing crypt depth or the villus-to-crypt ratio (*p* > 0.050). Jejunal villus height increased linearly with escalating doses of BCFAs-DFL (*p* < 0.001), whereas ileal villus height followed quadratic (*p* < 0.001) and cubic (*p* = 0.001) patterns. No significant interactions of treatment × sex were detected for any intestinal morphology parameters (*p* > 0.050).

### 3.6. Intestinal Microbiota

**Figure 4:**
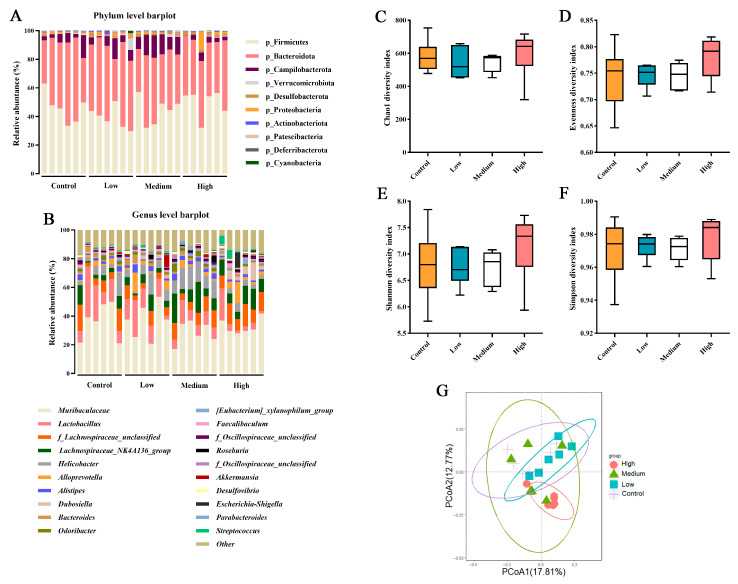
Effect of feeding different doses of BCFAs-DFL on intestinal microbiota. (**A**) Relative abundances of gut microbiota at the phylum level in the 4 groups. (**B**) Relative abundances of gut microbiota at the genus level in the 4 groups. (**C**) Analysis of alpha diversity of gut microbiota by Chao1 analysis. (**D**) Analysis of alpha diversity of gut microbiota by evenness analysis. (**E**) Analysis of alpha diversity of gut microbiota by Shannon analysis. (**F**) Analysis of alpha diversity of gut microbiota by Simpson analysis. (**G**) PCoA plots of beta diversity based on Bray–Curtis analysis in different groups. In this figure, n = 6 for each group. Each boxplot represents the median, interquartile range, minimum, and maximum values.

In this study, the dominant phyla and genera in the cecal microbiota of mice across all treatment groups exhibited similar compositions (Figure 4A,B). Administration of BCFAs-DFL at various doses did not induce significant alterations in α-diversity indices of the gut microbiota (*p* > 0.050; Figure 4C–F). Beta diversity analysis revealed high similarity among the control, low-, and medium-dose groups, with the high-dose group showing partial overlap with the medium-dose group (Figure 4G). For the differential abundance analysis, only taxa with a relative abundance above 0.05% at the genus level were included. The relative abundance of a total of 21 bacterial genera was significantly affected by the treatment (*p* ≤ 0.050; Table 7). No significant differences were observed between the low-dose and control groups (*p* > 0.050). In the medium-dose group, the relative abundance of *Faecalibaculum* was significantly lower than that in the control group, whereas *f_Desulfovibrionaceae_unclassified* and *Eubacterium_siraeum_group* showed the opposite trend (*p* ≤ 0.05). In the high group, the relative abundances of *Dubosiella*, *Escherichia-Shigella*, *Streptococcus*, *f_Desulfovibrionaceae_unclassified*, Lachnospiraceae_UCG-006, Oscillospiraceae_UCG-010, and *Eubacterium_siraeum_group* were significantly higher than those in the control group. Conversely, the relative abundances of *Odoribacter*, *Clostridia_vadinBB60_group*, *ASF356*, *Anaeroplasma*, and *Rikenellaceae* were significantly lower than those in the control group (*p* ≤ 0.05).

### 3.7. Spearman Correlation Analysis of Microbiota and Blood Parameters

**Figure 5:**
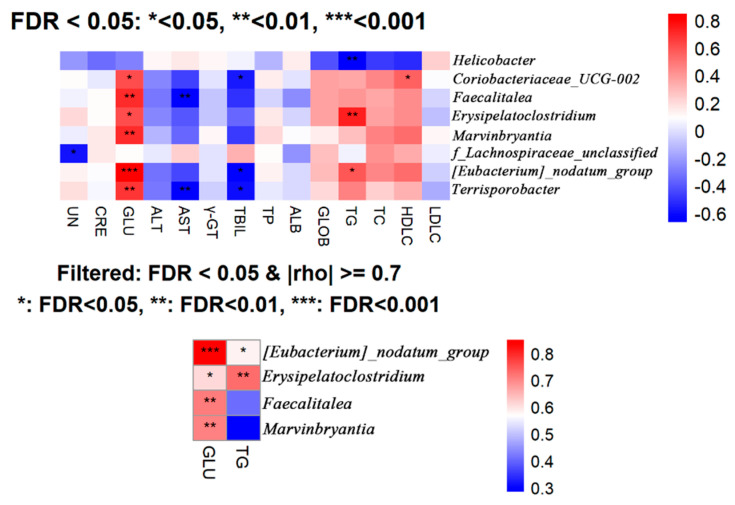
Spearman correlation analysis of microbiota and serum parameters. Serum parameters (TP = total protein, ALB = albumin, GLOB = globulin, AST = aspartate transaminase, ALT = alanine aminotransferase, TBIL = total bilirubin, TG = triglyceride, TC = total cholesterol, HDLC = high-density lipoprotein cholesterol, LDLC = low-density lipoprotein cholesterol, CRE = creatinine, UN = urea nitrogen, and GLU = glucose) were analyzed with significant different microbial genus. Color scale: red = positive correlation; blue = negative correlation.

Correlation analyses were conducted between significantly altered bacterial genera in mouse cecal contents and serum metabolites (Figure 5). The relative abundance of *f_Lachnospiraceae_unclassified* showed a significant negative correlation with blood UN levels (*p* < 0.05). Positive correlations with blood GLU were observed for Coriobacteriaceae_UCG-002, *Faecalitalea*, *Erysipelatoclostridium*, *Marvinbryantia*, *[Eubacterium]_nodatum_group*, and *Terrisporobacter* (*p* < 0.05). In contrast, *Faecalitalea* and *Terrisporobacter* were significantly negatively correlated with AST (*p* < 0.01). Blood TBIL levels were negatively associated with Coriobacteriaceae_UCG-002, *[Eubacterium]_nodatum_group*, and *Terrisporobacter* (*p* < 0.05). TG exhibited a negative correlation with *Helicobacter* but positive correlations with *Erysipelatoclostridium* and *[Eubacterium]_nodatum_group*. Additionally, Coriobacteriaceae_UCG-002 was positively correlated with HDLC (*p* < 0.05). GLU levels showed strong positive correlations (|ρ| > 0.7) with the relative abundances of *Erysipelatoclostridium*, *[Eubacterium]_nodatum_group*, *Faecalitalea*, and *Marvinbryantia*. Among these, *Erysipelatoclostridium* and *[Eubacterium]_nodatum_group* also exhibited strong correlations (|ρ| ≥ 0.7) with TG levels.

## 4. Discussion

This study represents the first investigation into the acute oral toxicity profiles of BCFAs-DFL. According to the OECD Guideline for the Testing of Chemicals 401 (OECD 401) issued in 1981, each dose group should include five animals per sex, and the LD50 (median lethal dose) has been integrated into a new assessment framework, with an upper dose limit of 5000 mg/kg body weight [31]. Under the OECD classification system for chemicals and mixtures, substances with an LD50 greater than 2000–5000 mg/kg are classified as unclassified or Category 5 and are considered to be of low toxicity [32]. In the present study, the acute toxicity test revealed no signs of poisoning, deaths, or significant behavioral changes among all mice. Additionally, parameters related to growth performance were unaffected by BCFAs-DFL treatment. These results suggest that BCFAs-DFL is safe at doses up to 5000 mg/kg.

In our investigation into the effects of BCFAs-DFL at doses of 100~600 mg/kg body weight on growth, metabolism, and immune function in mice, we observed that supplementation with 300 and 600 mg/kg BCFAs-DFL suppressed the ADFI, although it did not impair BW gain or ADG. Given that fatty acids themselves serve as energy substrates and prior studies have demonstrated that BCFAs can be absorbed and metabolized by tissues [33,34], this outcome is plausible. Moreover, iso-C17:0 and iso-C15:0 have been reported to act as growth regulators and nutritional factors that promote growth in Caenorhabditis elegans [35,36,37]. Taking into account the observed reduction in feed intake, we infer that BCFAs-DFL positively influences growth performance not only through nutritional regulation but also by enhancing feed digestion and absorption in mice. Changes in spleen and thymus weights are closely associated with immune function [38,39]. To date, no reference studies have addressed the impact of supplemental BCFAs on immunity. In the present study, BCFAs-DFL did not significantly alter spleen and thymus coefficients in mice; however, a dose-dependent trend was noted, implying potential immunomodulatory properties. Several studies indicate that spleen coefficients in mice correlate with variations in serum levels of IgA, IgG, and IgM [40,41]. IgA and IgG are critical for the establishment of the primary immune system and for protecting mucosal surfaces against toxins and pathogenic microorganisms [42,43]. The elevated serum IgA and IgG levels detected in BCFAs-DFL-supplemented mice may account for the dose-related increase in spleen coefficients. Elevated antibody levels can serve as an indicator of the immune system’s regulatory capacity; however, the actual regulatory effects of BCFAs on systemic immune function need to be further validated in disease challenge models in the future.

Hematological and biochemical parameters are widely employed to evaluate metabolic status and are influenced by dietary composition and environmental conditions [44,45]. RBC, HGB, and HCT are fundamental indicators of the body’s oxygen-carrying capacity and erythrocyte count [46]. High-dose BCFAs-DFL significantly reduced HGB and HCT levels, showing both linear and quadratic dose-dependent declines, which implies that 600 mg/kg BW may lower peripheral RBC counts and thereby diminish overall oxygen transport. Nevertheless, no significant change in RBC was detected in the high-dose group in this experiment. Previous reports also associate decreased HGB and HCT with anemia, trauma, immune modulation, and excessive water intake [47]. Additionally, high intake of fatty acids may induce metabolic reprogramming, leading to lipid and water retention to maintain osmotic equilibrium and facilitate excretion of metabolic wastes, potentially resulting in hemodilution and reduced HCT [48]. The impact of BCFAs-DFL on blood metabolism in mice remains poorly understood; however, feeding mice omega-3-rich fish oil yielded similar outcomes, and both BCFAs and omega-3 fatty acids share analogous roles in lipid metabolism regulation [49]. Serum ALB and GLOB levels displayed opposing trends, with GLOB significantly elevated in both low- and high-dose groups, possibly indicating altered hepatic synthesis or systemic inflammatory status [50]. This pattern of protein profile changes resembles that observed in certain prebiotic interventions with immunomodulatory effects, suggesting that BCFAs-DFL may modulate the immune-metabolic axis. The linear increase in AST and TBIL in medium- and high-dose groups indicates that high doses of BCFAs-DFL may impose a hepatic burden [51]. Moringa leaf extract, which shares anti-inflammatory, anticancer, and lipid-lowering properties with BCFAs, has similarly been shown to increase ALT and AST in mice at high doses (60~80 mg/kg), implying potential hepatotoxicity [52]. Therefore, we believe that the continuous supplementation of bioactive functional ingredients may impose a metabolic burden on the liver. TBIL, a hemoglobin breakdown product with antioxidant and anti-inflammatory activities, is negatively correlated with serum triglyceride levels [53]. In this study, BCFAs-DFL promoted TBIL production and showed an inverse relationship with TG levels. The significant reduction in HGB in the high-dose group may be attributable to accelerated hemoglobin catabolism into TBIL induced by BCFAs-DFL. Notably, administration at doses of 100–300 mg/kg BW led to a marked increase in serum TBIL levels, while blood HGB remained unaffected. In line with these findings, clinical investigations have further indicated that TBIL is capable of regulating lipid metabolism through the peroxisome proliferator-activated receptor α (PPARα) signaling pathway, a process that does not induce corresponding fluctuations in HGB concentrations [54,55,56]. Multiple studies have confirmed the lipid-lowering properties of BCFAs. In human blood and adipose tissues, the contents of various BCFAs such as iso-C15:0, anteiso-C15:0, iso-C16:0, and anteiso-C17:0 are negatively correlated with the concentration of triglycerides [57,58]. Furthermore, BCFAs derived from yak butter have been shown to suppress cholesterol synthesis in mouse plasma by modulating HMGCR gene expression [8]. This is consistent with the lower serum TC observed in female mice in the medium-dose group in our study. However, BCFAs-DFL appears to exert sex-specific effects on lipid metabolism. Free fatty acids stimulate hepatic gluconeogenesis, raising serum GLU [59]. In female mice, 600 mg/kg BCFAs-DFL increased TC, GLU, and AST levels, mirroring metabolic disturbances induced by high-fat diets [60,61]. However, the concurrent rise in HDLC contradicted this pattern. In humans, HDLC typically protects against atherosclerosis by promoting reverse cholesterol transport to the liver [62]. Thus, although higher fatty acid doses increase TC, BCFAs-DFL may counter this by elevating HDLC via its specific actions on lipid metabolism. In contrast, BCFAs-DFL only reduced serum TG and GLU in male mice, without affecting TC or HDLC, indicating that male mice tolerate higher doses of fatty acids than females.

An in vitro experiment revealed that multiple BCFAs (iso-C14:0, iso-C15:0, iso-C16:0, iso-C17:0, anteiso-C15:0, and anteiso-C17:0) inhibited the oxidative stress response of intestinal cells in calves caused by LPS and prevented the decrease in the contents of antioxidant enzymes such as CAT, T-AOC, and GSH-PX [63]. In this study, an increase in T-AOC levels was also observed. Serum MDA levels are established biomarkers of oxidative stress [64]. Zhang et al. [65] reported that BCFAs from sheep milk mitigated the DSS-induced decline in CAT and GSH in mouse blood. In this study, MDA, CAT, and GSH levels did not differ between the two treatment groups. Therefore, these results suggest that BCFAs-DFL supplementation can elevate the serum total antioxidant potential. Future studies employing in vivo oxidative stress challenge models will be required to elucidate whether the observed elevation in T-AOC can be translated into a functional protective effect against experimentally induced oxidative stress at the tissue level.

Intestinal cells exhibit specific absorption capacities for BCFAs-DFL, which also exerts strong preventive effects against intestinal inflammation [33,34,65]. Villi are key structures of the small intestine responsible for nutrient absorption, and villus height directly determines the intestinal absorptive surface area [66]. This study demonstrated that BCFAs-DFL promoted villus development in the jejunum and ileum of mice. Therefore, we propose that BCFAs-DFL enhances intestinal architecture and nutrient absorption efficiency, which is consistent with the improved growth performance observed in mice.

Based on the results of the microbial composition and diversity in the cecal contents of mice, it can be concluded that supplementation with BCFAs-DFL at different doses has minimal impact on the microbial diversity and major community structure of the mouse cecal microbiota. Alpha diversity describes the richness, evenness, or overall diversity of microbial species within a sample, while beta diversity is used to compare the similarities between two or more communities [67]. The findings of this study indicate that BCFAs-DFL did not alter the richness or evenness of the gut microbiota across the three doses (Figure 4C–F). Furthermore, there was high similarity among the control, low-dose, and medium-dose groups, with only partial overlap between the high-dose group and the medium-dose group (Figure 4G). It can be inferred that supplementation of up to 600 mg/kg body weight with BCFAs-DFL may induce some differences in the gut microbial community structure. The analysis at the genus level revealed that the low-dose BCFAs-DFL group showed no significant differences compared to the control group, which is consistent with the beta diversity results. Results from the present study indicate that dietary supplementation with BCFAs-DFL specifically modulates the abundance of certain bacterial taxa without markedly altering the overall structure of the cecal microbiota in mice, thereby inducing systemic changes associated with host metabolism and health. This finding aligns with previous research suggesting that certain dietary interventions tend to regulate key groups within the ecosystem rather than trigger drastic shifts in community structure [68]. Notably, BCFAs-DFL supplementation significantly influenced several bacterial groups closely linked to host health. For example, the relative abundance of *Dubosiella* exhibited a dose-dependent increase. This genus has been reported to contribute to improved host metabolism and enhanced intestinal barrier function via the production of short-chain fatty acids such as acetate [69]. Among the bacterial genera altered by BCFA treatment, *Eubacterium_siraeum_group* has been shown to ferment disaccharides into acetate, convert bile acids and cholesterol in the gut, and thereby contribute to host metabolic homeostasis [70,71]. Consistently, it has been reported to correlate negatively with serum levels of TG and total cholesterol [72], a trend also observed in the present study—particularly at a dose of 300 mg/kg BW—though this specific correlation was not captured in our overall analysis and warrants further investigation. Other modulated genera include *Rikenellaceae*, which may promote the reduction in visceral adipose tissue and the generation of beneficial metabolites [73], as well as *Streptococcus* and *Escherichia-Shigella*, which are often linked to intestinal pathogenicity and a pro-inflammatory state [74,75]. Within the dosage range of 100–300 mg/kg BW, the relative abundance of most microbial genera remained relatively stable; however, the high-dose group (600 mg/kg BW) exhibited marked shifts in certain beneficial and pathogenic taxa. Considering the concurrent significant changes in blood metabolic markers, we speculate that at this higher dose, BCFAs might induce a degree of metabolic disturbance. Moreover, the majority of the significantly altered genera identified here are consistently associated in the literature with intestinal health or lipid metabolism, aligning with the previously reported bioactivity of BCFAs. After performing correlation analysis between microbial relative abundance and blood biochemical parameters, we identified several bacterial genera associated with glucose and lipid metabolism indicators. Among these, *Erysipelatoclostridium*—a genus previously linked to pro-inflammatory responses—showed a positive correlation with TG level [76], which aligns with the findings presented here. It should be noted that the present work represents a preliminary exploration of BCFA functionality and dosage, and the sample size used for microbial profiling was relatively limited. While the observed correlations offer insightful directions, the current scale may not be sufficient to firmly establish their physiological relevance. Therefore, these results are presented as indicative findings that highlight potential regulatory mechanisms of BCFAs. In subsequent studies, extending the experimental timeline and expanding the sample size will allow for a more detailed and statistically robust investigation of the gut microbiota and its metabolic interactions.

## 5. Conclusions

Based on the findings of this study, the preliminary acute tolerance test and 28-day intervention results indicate that BCFAs-DFL show no obvious acute or short-term adverse reactions in mice at doses up to 5000 mg/kg BW. The 28-day intervention results reveal preliminary biological effects of BCFAs-DFL on host physiology: they can modulate host metabolism by decreasing serum triglycerides, enhancing T-AOC and immunoglobulin levels, and promoting the development of intestinal villi. However, adverse effects were also observed at specific doses: BCFAs-DFL at doses ranging from 300~600 mg/kg BW increased serum aspartate AST levels, which may impose a certain burden on liver metabolism; at 600 mg/kg BW, they may significantly increase the risk of intestinal microbial structural disorder, possibly associated with the elevated AST level. Therefore, in the short-term study, a dose of 100 mg/kg BW can be used as a reference.

## Figures and Tables

**Table 1:** Composition and content of branched-chain fatty acids derived from lanolin (BCFAs-DFL).

Fatty Acids ^1^	Content (% Total Fatty Acids) ^2^
*iso*-C10:0	0.77 ± 0.05
C10:0	0.83 ± 0.09
*anteiso*-C11:0	2.66 ± 0.11
C11:0	0.17 ± 0.02
*iso*-C12:0	3.83 ± 0.17
C12:0	1.02 ± 0.01
*iso*-C13:0	0.45 ± 0.01
*anteiso*-C13:0	4.94 ± 0.16
C13:0	0.57 ± 0.05
*iso*-C14:0	14.47 ± 0.72
C14:0	5.49 ± 0.68
*iso*-C15:0	2.71 ± 0.02
*anteiso*-C15:0	16.56 ± 0.12
C15:0	1.52 ± 0.20
*iso*-C16:0	14.27 ± 0.19
C16:0	2.73 ± 0.53
C16:1	0.74 ± 0.06
*iso*-C17:0	0.70 ± 0.03
*anteiso*-C17:0	8.22 ± 0.32
*iso*-C18:0	5.28 ± 0.44
C18:0	1.45 ± 0.10
C18:1	0.66 ± 0.08
*anteiso*-C19:0	6.00 ± 0.58
*iso*-C20:0	2.14 ± 0.31
*anteiso*-C21:0	1.83 ± 0.33
*iso*-BCFAs	44.62 ± 0.50
*anteiso*-BCFAs	40.20 ± 0.89
total BCFAs	84.82 ± 1.22

^1^ *iso*-C10:0 = 8-methylnonanoic acid; C10:0 = decanoic acid; *anteiso*-C11:0 = methyl 8-methyl-decanoate; C11:0 = undecanoic acid; *iso*-C12:0 = 10-methylundecanoic acid; C12:0 = dodecanoic acid; *iso*-C13:0 = 11-methyldodecanoic acid; *anteiso*-C13:0 = 10-methyldodecanoic acid; C13:0 = tridecanoic acid; *iso*-C14:0 = 12-methyltridecanoic acid; C14:0 = myristic acid; *iso*-C15:0 = 13-methyltetradecanoic acid; *anteiso*-C15:0 = 12-methyltetradecanoic acid; C15:0 = pentadecanoic acid; *iso*-C16:0 = 14-methylpentadecanoic acid; C16:0 = hexadecanoic acid; C16:1 = 9-hexadecenoic acid; *iso*-C17:0 = 15-methylhexadecanoic acid; *anteiso*-C17:0 = 14-methylhexadecanoic acid; *iso*-C18:0 = isostearic acid; C18:0 = stearic acid; C18:1 = 9-octadecenoic acid; *anteiso*-C19:0 = 16-methyloctadecanoic acid; *iso*-C20:0 = 18-methylnonadecanoic acid; *anteiso*-C21:0 = 18-methyleicosanoic acid. ^2^ Data is presented as mean ± SD.

**Table 2:** Death results of the acute toxicity test of BCFAs-DFL.

Sex	Treatments	Dose (mg/kg·BW)	Size of Animal	Approach	Deaths
Female	Control	0	5	Administered orally	0
	BCFA	5000	5	Administered orally	0
Male	Control	0	5	Administered orally	0
	BCFA	5000	5	Administered orally	0

**Table 3:** Effect of BCFAs-DFL on growth performance and immune organ coefficients of mice.

Items ^1^	Treatment ^2^				SEM	Effect (*p*-Value)			Contrast (*p*-Value)		
	CON	Low	Medium	High		Treat	Sex	Treat × Sex	Linear	Quadratic	Cubic
Growth performance											
BW of d 0, g	16.30	16.73	16.47	16.72	0.290	0.678	<0.001	0.808	0.510	0.923	0.307
BW of d 28, g	18.95	19.54	18.72	18.92	0.273	0.190	<0.001	0.976	0.389	0.834	0.048
ADG, g/d	0.09	0.10	0.08	0.08	0.006	0.063	<0.001	0.535	0.023	0.636	0.157
ADFI, g/d	2.81 ^a^	2.91 ^a^	2.59 ^b^	2.63 ^b^	0.040	<0.001	0.319	0.953	<0.001	0.071	0.001
Organ coefficients, % BW											
Spleen	0.33	0.36	0.34	0.37	0.013	0.094	<0.001	0.078	0.115	0.710	0.049
Thymus	0.25	0.26	0.23	0.21	0.018	0.168	<0.001	0.377	0.036	0.791	0.476

^a,b^ Means within a row with different letters are significantly different (*p* < 0.050). ^1^ ADG = (BW of d 28 − BW of d 0)/28; ADFI = average daily feed intake. ^2^ CON = control; low = 100 mg/kg/day BCFAs-DFL; medium = 300 mg/kg/day BCFAs-DFL; high = 600 mg/kg/day BCFAs-DFL.

**Table 4:** Effect of BCFAs-DFL on hematology indexes in mice.

Items	Treatment ^1^				SEM	Effect (*p*-Value)			Contrast (*p*-Value)		
	CON	Low	Medium	High		Treat	Sex	Treat × Sex	Linear	Quadratic	Cubic
WBC, 10^9^/L	6.61	6.88	6.47	6.58	0.246	0.684	<0.001	0.220	0.610	0.854	0.280
LYM, 10^9^/L	5.06	5.04	4.7	5.21	0.198	0.332	<0.001	0.203	0.683	0.106	0.443
RBC, 10^12^/L	8.50	8.51	8.51	8.20	0.095	0.075	0.026	0.612	0.023	0.187	0.797
HGB, g/L	104.50 ^a^	106.60 ^a^	106.40 ^a^	100.40 ^b^	1.175	0.002	0.146	0.745	0.004	0.007	0.697
HCT, %	39.48 ^a^	39.22 ^a^	39.64 ^a^	36.74 ^b^	0.553	0.002	0.497	0.764	0.001	0.043	0.362
MCV, fL	46.46 ^ab^	46.79 ^a^	46.46 ^ab^	46.01 ^b^	0.172	0.028	0.039	0.862	0.013	0.180	0.212
PCT, %	0.374 ^ab^	0.400 ^a^	0.371 ^b^	0.348 ^b^	0.009	0.006	0.234	0.938	0.005	0.194	0.049
GRA, 10^9^/L	3.76	3.66	3.70	3.66	0.148	0.958	<0.001	0.434	0.736	0.884	0.682

^a,b^ Means within a row with different letters are significantly different (*p* < 0.050). ^1^ CON = control; low = 100 mg/kg/day BCFAs-DFL; medium = 300 mg/kg/day BCFAs-DFL; high = 600 mg/kg/day BCFAs-DFL.

**Table 5:** Effect of BCFAs-DFL on serum metabolites in mice.

Items	Treatment ^1^				SEM	Effect (*p*-Value)			Contrast (*p*-Value)		
	CON	Low	Medium	High		Treat	Sex	Treat × Sex	Linear	Quadratic	Cubic
Biochemical parameter											
TP, g/L	57.99	57.19	56.38	55.52	0.925	0.286	0.736	0.074	0.060	0.715	0.881
ALB, g/L	35.55 ^a^	33.40 ^b^	34.65 ^ab^	31.11 ^c^	0.624	<0.001	0.055	0.072	<0.001	0.247	0.011
GLOB, g/L	22.44 ^b^	23.79 ^ab^	21.73 ^bc^	24.41 ^a^	0.520	0.004	0.083	0.293	0.067	0.047	0.006
ALT, U/L	38.20	37.90	40.05	38.68	0.906	0.363	0.714	0.981	0.467	0.223	0.279
AST, U/L	42.26 ^c^	43.43 ^c^	50.21 ^b^	56.58 ^a^	1.422	<0.001	0.401	0.006	<0.001	0.793	0.369
γ-GT, U/L	2.30	2.40	3.55	3.26	0.562	0.317	0.867	0.758	0.146	0.327	0.502
TBIL, μmol/L	2.19 ^c^	2.69 ^b^	2.67 ^b^	3.14 ^a^	0.121	<0.001	0.065	0.018	<0.001	0.602	0.035
TG, mmol/L	1.31 ^a^	0.91 ^b^	0.70 ^c^	1.05 ^b^	0.062	<0.001	0.485	0.024	0.048	<0.001	0.271
TC, mmol/L	2.06 ^ab^	2.09 ^ab^	1.91 ^b^	2.28 ^a^	0.084	0.034	0.071	0.048	0.097	0.030	0.200
HDLC, mmol/L	1.60 ^ab^	1.66 ^a^	1.49 ^b^	1.73 ^a^	0.055	0.026	0.003	0.010	0.192	0.034	0.057
LDLC, mmol/L	0.29	0.30	0.32	0.25	0.017	0.067	0.179	0.488	0.172	0.024	0.556
CRE, mg/dL	2.33	2.03	2.23	1.87	0.212	0.432	0.352	0.443	0.222	0.786	0.282
BUN, mmol/L	7.33 ^a^	6.36 ^ab^	5.49 ^b^	5.36 ^b^	0.366	0.002	0.281	0.273	0.001	0.049	0.729
GLU, mg/dL	2.93	2.81	2.53	2.63	0.257	0.690	0.013	<0.001	0.371	0.439	0.841
Antioxidant parameter											
CAT, U/mL	29.22 ^ab^	34.46 ^a^	24.93 ^b^	24.84 ^b^	2.064	0.007	0.004	0.067	0.012	0.797	0.011
GSH-PX, U/mL	554.72	581.32	613.62	537.63	20.940	0.077	<0.001	0.195	0.469	0.013	0.766
T-AOC, U/mL	27.87 ^c^	36.01 ^b^	37.00 ^b^	51.55 ^a^	2.776	<0.001	0.049	0.194	<0.001	0.645	0.117
MDA, mmol/mL	32.57	31.15	30.09	32.92	2.488	0.842	0.164	0.582	0.853	0.379	1.000
Immunoglobulin											
IgA, μg/mL	0.44 ^c^	0.58 ^a^	0.57 ^a^	0.51 ^b^	0.014	<0.001	0.098	0.573	0.097	<0.001	<0.001
IgG, μg/mL	2.01 ^c^	2.62 ^b^	3.43 ^a^	2.48 ^b^	0.140	<0.001	0.917	0.561	0.034	<0.001	0.479
IgM, ng/mL	34.08	37.12	34.75	36.48	1.376	0.371	0.848	0.414	0.514	0.965	0.104

^a~c^ Means within a row with different letters are significantly different (*p* < 0.050). ^1^ CON = control; Low = 100 mg/kg/day BCFAs-DFL; medium = 300 mg/kg/day BCFAs-DFL; high = 600 mg/kg/day BCFAs-DFL.

**Table 6:** Effects of BCFAs-DFL supplementation on mice intestinal development.

Items ^1^	Treatment ^2^				SEM	Effect (*p*-Value)			Contrast (*p*-Value)		
	CON	Low	Medium	High		Treat	Sex	Treat × Sex	Linear	Quadratic	Cubic
Jejunum											
Villus height, µm	292.09 ^c^	305.64 ^b^	311.36 ^b^	322.14 ^a^	2.810	<0.001	0.196	0.059	<0.001	0.121	0.104
Crypt depth, µm	78.54	80.23	79.02	80.47	3.192	0.967	0.588	0.909	0.764	0.970	0.692
V/C	3.83	3.95	3.97	4.14	0.168	0.634	0.506	0.687	0.221	0.999	0.719
Ileum											
Villus height, µm	192.56 ^c^	228.19 ^a^	228.07 ^a^	207.44 ^b^	3.281	<0.001	0.381	0.274	0.291	<0.001	0.001
Crypt depth, µm	74.92	85.39	86.18	84.01	3.767	0.168	0.626	0.362	0.226	0.103	0.294
V/C	2.60	2.70	2.72	2.58	0.122	0.787	0.648	0.274	0.789	0.347	0.826

^a~c^ Means within a row with different letters are significantly different (*p* < 0.050). ^1^ V/C = Villus height/crypt depth. ^2^ CON = control; low = 100 mg/kg/day BCFAs-DFL; medium = 300 mg/kg/day BCFAs-DFL; high = 600 mg/kg/day BCFAs-DFL.

**Table 7:** Effect of BCFAs-DFL supplementation on the bacterial genus (relative abundances > 0.05%) in the cecal contents of mice.

Taxonomic Level	Treatment ^1^				SEM	*p*-Value (FDR)			
	CON	Low	Medium	High		Overall	C vs. L	C vs. M	C vs. H
*Helicobacter*	6.13	6.68	12.45	3.24	0.209	0.011	0.995	0.196	0.089
*Dubosiella*	0.41	1.34	0.28	4.98	0.115	<0.001	0.544	0.464	0.003
*Odoribacter*	1.61	1.10	2.90	0.53	0.056	<0.001	0.875	0.306	0.003
*Faecalibaculum*	1.24	1.07	0.33	1.08	0.027	0.048	0.977	0.025	0.702
*f_Oscillospiraceae_uncultured*	0.71	0.48	1.34	0.69	0.029	0.029	0.937	0.103	0.953
*Escherichia-Shigella*	0.14	0.39	0.13	2.24	0.090	<0.001	0.636	0.938	<0.001
*Streptococcus*	0.19	0.43	0.11	2.00	0.063	<0.001	0.636	0.464	0.002
*Colidextribacter*	0.42	0.30	0.65	0.69	0.015	0.032	0.833	0.218	0.127
*f_Desulfovibrionaceae_unclassified*	0.21	0.46	0.64	0.69	0.013	0.011	0.219	0.021	0.003
*Blautia*	0.41	0.46	0.22	0.66	0.015	0.050	0.937	0.103	0.601
*Lachnospiraceae_UCG-006*	0.17	0.24	0.46	0.76	0.019	0.012	0.833	0.122	0.003
*Clostridia_vadinBB60_group*	0.38	0.32	0.58	0.09	0.013	<0.001	0.937	0.556	<0.001
*Eubacterium_coprostanoligenes_group*	0.43	0.41	0.18	0.23	0.011	0.042	0.937	0.066	0.065
*ASF356*	0.40	0.21	0.18	0.20	0.008	0.050	0.499	0.076	0.049
*Anaeroplasma*	0.16	0.38	0.36	0.05	0.010	0.001	0.499	0.343	0.038
*Rikenellaceae*	0.28	0.24	0.24	0.10	0.007	0.029	0.937	0.464	0.004
*Coriobacteriaceae_UCG-002*	0.15	0.48	0.07	0.14	0.016	<0.001	0.190	0.185	0.570
*f_Lachnospiraceae_unclassified*	0.11	0.10	0.09	0.24	0.004	0.034	0.998	0.808	0.065
*Mucispirillum*	0.09	0.10	0.28	0.05	0.005	0.030	0.937	0.119	0.367
*Oscillospirales_UCG-010*	0.06	0.10	0.07	0.24	0.004	0.011	0.636	0.980	0.010
*Eubacterium_siraeum_group*	0.05	0.08	0.19	0.14	0.005	0.038	0.544	0.021	0.035

^1^ CON = control; low = 100 mg/kg/day BCFAs-DFL; medium = 300 mg/kg/day BCFAs-DFL; high = 600 mg/kg/day BCFAs-DFL.

## Data Availability

The original contributions presented in this study are included in the article. Further inquiries can be directed to the corresponding authors.

## Abbreviations

The following abbreviations are used in this manuscript:

ANOVA	Analysis of variance
BCFAs-DFL	Branched-chain fatty acids derived from lanolin
H&E	Hematoxylin and eosin
RBC	Red blood cells
MCV	Mean corpuscular volume
HCT	Hematocrit
PCT	Platelet crit
WBC	White blood cells
LYM	Lymphocytes
GRA	Granulocytes
HGB	Hemoglobin
TP	Total protein
ALB	Albumin
GLOB	Globulin
AST	Aspartate transaminase
ALT	Alanine aminotransferase
TBIL	Total bilirubin
TG	Triglyceride
TC	Total cholesterol
HDLC	High-density lipoprotein cholesterol
LDLC	Low-density lipoprotein cholesterol
CRE	Creatinine
BUN	Blood urea nitrogen
GLU	Glucose
